# Neuromodulation for Restless Legs Syndrome: A Systematic Review and Mechanistic Considerations for Spinal Cord Stimulation

**DOI:** 10.1007/s11325-026-03638-7

**Published:** 2026-03-10

**Authors:** Garrett W. Thrash, Elijah Wang, David Brockington, Colby Rives, Yifei Sun, Anne C. Roberts, Somnath Das, Julie G. Pilitsis, Prasad Shirvalkar, Rocio Vazquez do Campo, Harrison C. Walker, Christopher J. Earley, Marshall T Holland

**Affiliations:** 1 Heersink School of Medicine, University of Alabama at Birmingham, Birmingham, AL USA; 2 Department of Neurosurgery, University of Alabama at Birmingham, FOT Suite 1060, 1720 2nd Ave S, Birmingham, AL 35294 USA; 3 Department of Neurosurgery, University of Arizona, Tucson, AZ USA; 4 Departments of Neurological Surgery, Anesthesiology and Neurology, University of California San Francisco, San Francisco, CA USA; 5 Department of Neurology, University of Alabama at Birmingham, Birmingham, AL USA; 6 Department of Neurology, Johns Hopkins University School of Medicine, Baltimore, MD USA

**Keywords:** Neuromodulation, Restless Legs Syndrome, Spinal Cord Stimulation, Deep Brain Stimulation, Transcranial Magnetic Stimulation

## Abstract

**Background:**

Restless legs syndrome (RLS) is a common sleep disorder characterized by an irresistible urge to move the legs. Symptoms predominate in the evening and cause significant circadian rhythm interruptions that impact sleep and quality of life, leading to decreased work productivity, anxiety, and depression. Some patients improve with oral medications, but many experience treatment-refractory symptoms, whether from lack of efficacy or intolerable cognitive or behavioral side effects. Emerging evidence suggests that diverse neuromodulation modalities could provide a novel circuit-based therapy option for patients with treatment-refractory RLS. Given its prevalence, most of these reports arise as serendipitous observations of improved RLS symptoms when it is comorbid with an approved indication for neuromodulation, such as chronic neuropathic pain or a movement disorder. More recent work has begun investigating neuromodulation, including spinal cord stimulation, for primary idiopathic RLS.

**Methods:**

Here we performed a PRISMA systematic review identifying 120 articles utilizing spinal cord stimulation, deep brain stimulation, transcranial magnetic stimulation, vagal nerve stimulation, and other modalities. Among these, we summarize findings from 54 studies that met our specific inclusion criteria and suggest potential mechanisms, such as spinal cord hyperexcitability, for neuromodulation of RLS symptoms.

**Results:**

Interestingly, the amount and duration of RLS improvement seems to correlate with the invasiveness of the neuromodulation modality. Spinal cord stimulation has demonstrated more consistent, promising results with greater magnitude of IRLS Score improvement compared to the other modalities.

**Conclusion:**

Prospective, multi-institutional trials are needed to evaluate whether various neuromodulation strategies can impact debilitating symptoms from treatment-refractory RLS, and rigorous analysis should be conducted to identify the most effective modalities.

## Introduction

Restless legs syndrome (RLS) disrupts sleep with patients characteristically experiencing an unrelenting urge to move the affected limb with voluntary limb movements providing only temporary relief. These symptoms show a circadian rhythm, peaking in the evening and during sleep. The disruption of restful sleep causes excessive daytime sleepiness, loss of productivity, depression, and anxiety. This additionally yields increased rates of morbidity and mortality from hypertension, stroke, renal and cardiac disease [1]. With up to 30 million US citizens suffering from RLS and/or its treatments, RLS is the most common sleep disorder [2].

Iron deficiency occurs in 25–40% of patients with RLS, and iron supplementation is the first-line treatment [3]. Standard oral medications include dopaminergic agonists (e.g., ropinirole, pramipexole, rotigotine) and ⍺2δ calcium channel ligands (gabapentin, pregabalin) [4]. Patients with severe or treatment-refractory symptoms often experience augmentation, requiring repeated dose escalations and can experience cognitive and/or behavioral side effects from polypharmacy. This leads most RLS patients to discontinue all standard medication therapies by 10 years, leaving them with chronic, distressing symptoms, an inadequate quality of life (QoL), and a paucity of viable treatment options [5]. Opioids may be utilized in treatment-refractory cases, but this option is associated with social stigma, addiction concerns, and increasing regulatory constraints [2,3]. This leaves many patients with treatment-refractory RLS symptoms that negatively impact their social, occupational, psychological, and medical function.

RLS pathophysiology is unclear, but evidence suggests widespread nervous system hyperexcitability including the basal ganglia, cortex, and spinal cord (SC), and increased sympathetic tone [6]. There are no widely accepted animal model systems for this disorder because animals cannot report urges. This uncontrollable sensation in the legs is highly irritating but typically not painful with patients commonly describing a deep sensation (not on the skin surface) that is temporarily relieved by voluntary leg muscle contractions [7]. Afferents from skeletal muscles and tendons provide proprioceptive information and travel via large, fast-conducting Aα fibers through the dorsal column of the SC [8]. RLS may be associated with SC hyperexcitability given abnormalities noted in the H-reflex [9–16]. Abnormal cortical excitability is suggested by reduced transcranial magnetic stimulation (TMS) motor thresholds, prolonged cutaneous silent periods, and symptom improvement, albeit temporarily, with repetitive TMS (rTMS) [9,17–24].

**Fig. 1:**
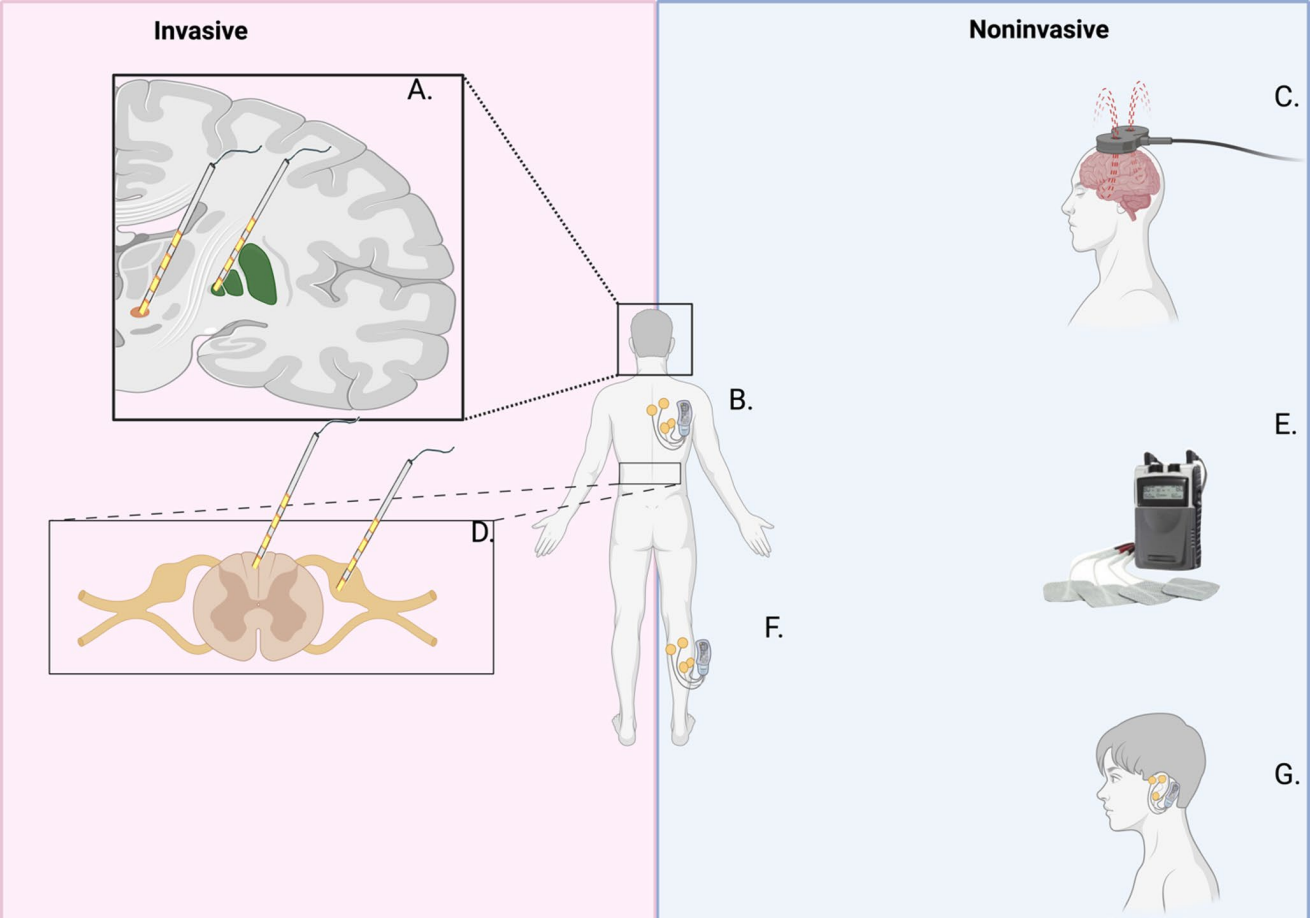
Illustration of Neuromodulatory modalities to treat RLS. **A** Deep brain stimulation (DBS) in the STN (orange) and GPi of the lentiform nucleus (Green). **B** Transcutaneous spinal direct current stimulation (tsDCS). **C** Transcranial magnetic stimulation (TMS). **D** Dorsal root ganglion (DRG) stimulation and spinal cord stimulation (SCS). **E** Transcutaneous electric stimulation (TENS) of the affected leg. **F** Noninvasive peripheral nerve stimulation (NPNS) of the peroneal nerve. **G** TVNS using an earpiece

Neuromodulation for RLS was first investigated in a 1999 case series that utilized TMS [25]. Subsequent studies have examined more invasive modalities including spinal cord stimulation (SCS), deep brain stimulation (DBS), and peripheral neuromuscular stimulation. The gold standard measure of RLS severity is the validated International Restless Legs Syndrome Rating Scale (IRLS Score) where a reduction of ≥ 3 points is considered clinically significant [26, 27]. Evidence from these neuromodulation studies suggests potential effectiveness of circuit-based therapies that avoid lack of systemic side effects from oral medications. In this review, we summarize the history and findings from prior studies of neuromodulation therapies for treatment-refractory RLS and suggest pathophysiological mechanisms. We consider this review necessary to pinpoint treatment methodologies that have shown to be the least and most effective to increase the universal precision of treatment of the disorder (Fig. 1).

## Methods

We performed a systematic review of the literature on 09/07/2024 in PubMed using PRISMA guidelines included in the supplement for the following search terms:

(“Restless legs syndrome“[tiab] OR “Restless legs syndrome“[MeSH] OR “RLS“[tiab]) AND (“Deep brain stimulation“[tiab] OR “DBS” [tiab] OR “Spinal cord stimulation“[tiab] OR “SCS“[tiab] OR “Neuromodulation“[Title/Abstract] OR “transcranial magnetic stimulation“[Title/Abstract] OR “TMS“[Title/Abstract])

**Fig. 2:**
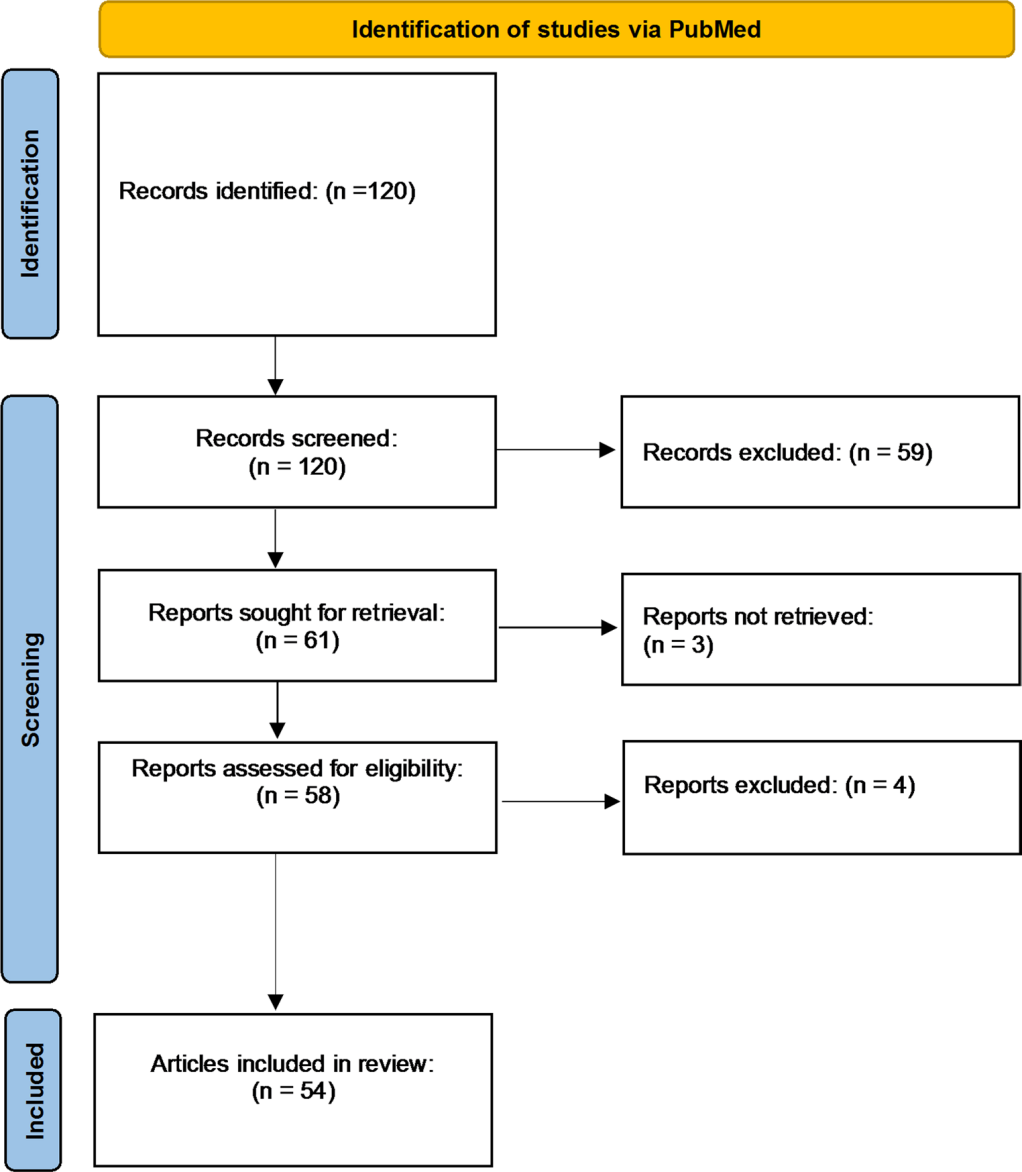
PRISMA flow diagram from PubMed literature search

120 articles were exported and screened independently by authors G.T., A.R. and E.W. using Covidence. All conflicts were resolved by M.H. Inclusion criteria were (1) original article that involved (2) neuromodulation of RLS, and that (3) suggested potential mechanisms for RLS. All reviews, commentaries, and other reports on the articles were excluded. We found 54 primary unique articles for the neuromodulation of RLS and summarized their findings in this review in U.S. English (Fig. 2). The review protocol was not registered in a database.

A risk-of-bias assessment was performed using the Methodological Index for Non-Randomized Studies (MINORS) scale (Table 1). Scores were assigned in 12 unique categories on a scale from 0 to 2 with a potential maximum score of 24. A higher resulting score is correlated with a lower risk of bias.

**Table 1:** Risk-of-bias assessment using the MINORS scale to assess quality of studies

Study	A clearly stated aim	Inclusion of consecutive samples	Prospective collection of data	Endpoints appropriate to the aim of the study	Unbiased assessment of the study endpoint	Assessment tests appropriate with the aim	Loss of samples < 5%	Prospective calculation of the study size	An adequate control group	Contemporary groups	Baseline equivalence of groups	Adequate statistical analyses	Results
Evidente et al.	2	2	1	2	0	2	2	2	2	1	2	2	20
Ondo et al.	0	2	1	2	0	2	2	2	0	0	2	0	13
Okun et al.	0	2	1	2	0	0	0	2	0	0	2	0	9
Holland et al.	1	2	1	2	0	2	2	2	0	0	2	0	14
Adil et al.	2	2	1	2	0	1	2	2	0	0	2	0	14
Byrne et al.	1	2	1	2	0	1	2	2	0	0	2	0	13
De Vloo et al.	1	2	1	2	0	2	2	2	0	0	2	0	14
Klepitskaya et al.	2	2	1	2	0	1	1	1	0	0	2	2	14
Chahine et al.	2	2	2	2	0	1	2	1	1	0	2	2	17
Driver-Dunckley et al.	1	2	1	2	0	1	2	1	0	0	2	2	14
Dulski et al.	2	2	2	2	0	1	2	1	2	2	2	2	20
Hidding et al.	2	2	2	2	2	0	0	1	0	0	2	2	15
Kedia et al.	2	2	1	2	0	0	0	1	0	0	2	2	12
Lei et al.	2	2	1	2	0	1	2	2	2	2	2	2	20
Marques et al.	2	2	2	2	0	1	2	1	0	0	2	2	16
Tordjman et al.	0	2	1	2	0	0	0	2	0	0	2	2	11
Tolleson et al.	2	2	2	2	0	0	0	1	0	0	2	2	13
Abd-Elsayed et al.	2	2	1	2	0	1	2	2	0	0	2	0	14
Altunrende et al.	2	2	2	2	2	0	0	2	2	2	2	2	20
Ahlgren-Rimpilainen et al.	2	2	2	2	0	0	0	2	2	2	2	2	18
Bocquillon et al.	2	2	2	2	0	0	0	2	2	2	2	2	18
Bogan et al.	2	2	2	2	2	1	2	2	2	2	2	2	23
Buchfuhrer et al.	2	2	1	2	0	0	0	2	2	2	2	2	17
Buchfuhrer et al.	2	2	2	2	0	0	0	2	0	0	2	2	14
Casoni et al.	0	2	1	2	0	2	2	2	0	0	2	0	13
de Paiva et al.	2	2	1	2	2	2	2	1	2	2	2	2	22
Gorsler et al.	2	2	1	2	0	0	0	2	0	0	2	2	13
Gunduz et al.	2	2	1	2	0	0	0	2	2	2	2	2	17
Hackethal et al.	0	2	1	2	0	1	2	2	0	0	2	2	14
Hartley et al.	2	2	1	2	0	0	0	2	0	0	2	2	13
Klung et al.	1	2	1	2	0	0	0	2	0	0	2	2	12
Kutukcu et al.	2	2	1	2	0	0	0	1	2	2	2	2	16
Lanza et al.	2	2	1	2	0	0	0	0	2	2	2	2	15
Lin et al.	2	2	1	2	0	0	0	2	0	0	2	2	13
Lin et al.	2	2	1	2	0	0	0	2	2	2	2	2	17
Liu et al.	2	2	1	2	0	0	0	1	2	2	1	2	15
Magalhaes et al.	2	2	1	2	1	0	0	1	2	2	2	2	17
Ondo et al.	1	2	1	2	0	2	2	2	0	0	2	0	14
Quatrale et al.	2	2	1	2	0	0	0	2	2	2	2	1	16
Rizzo et al.	2	2	1	2	0	1	1	2	2	2	2	2	19
Rizzo et al.	2	2	1	2	0	1	1	2	2	2	2	2	19
Roy et al.	2	2	2	2	2	0	0	1	2	2	2	2	19
Salas et al.	2	2	1	2	0	0	0	2	2	2	2	2	17
Sanli et al.	2	2	2	2	1	1	2	2	2	2	2	2	22
Scalise et al.	2	1	2	2	0	0	0	2	0	0	2	2	13
Scalise et al.	2	1	1	2	0	0	0	2	2	2	2	2	16
Singh et al.	2	2	2	2	1	0	0	2	2	2	2	2	19
Tergau et al.	2	1	1	2	0	0	0	2	2	2	2	2	16
Zeng et al.	2	2	1	2	0	0	0	2	2	2	2	2	17

## Results

### Noninvasive peripheral nerve stimulation

Noninvasive peripheral nerve stimulation (NPNS) of the peroneal nerve, also known as tonic motor activation (TOMAC), represents one of the newest low-invasive modulatory modalities for RLS. NPNS works through wearable, bilateral external stimulators fitted over the fibula that engage similar proprioceptive and sensory circuitry thought to suppress RLS symptoms as leg movements or walking do [28]. Multiple trials provided evidence of NPNS being a safe and efficacious treatment of RLS through subjective reporting of clinical global impressions-improvement (CGI-I) and IRLS scores [28–32]. One study demonstrated a decrease in opioid use, which can be used as medical therapy for RLS refractory to front-line treatment. However, NPNS stimulation provided only a small magnitude of decrease seen in IRLS scores with the mean differences of 3.4, 4.4, 7.4, and 11.3 decreased scores reported [28–32]. While any improvement can be seen as a positive therapy, this does not broach the magnitude of difference in RLS scores seen in some of the SCS and DBS case reports [33]. However, comparison of symptoms across studies and modalities carries no statistical significance due to heterogeneous reporting, highlighting the call for rigorous reporting guidelines and further clinical trials. This highlights NPNS as a non-invasive and efficacious, albeit mild, treatment for RLS.

### Noninvasive transauricular vagus nerve stimulation

Noninvasive transauricular vagus nerve stimulation (tVNS) for treatment-refractory RLS has also been studied via a clinical trial [34]. In this study, 66% of patients saw improvement in symptoms (as seen in NPNS). However, the mean decrease in IRLS scores was 7. Importantly, this trial also demonstrated improvements in QoL, anxiety, and depression. This trial highlights the importance of measures beyond RLS symptom severity and sleep disturbance.

### Transcranial magnetic stimulation

TMS has a long record of accomplishment as a diagnostic and monitoring tool of RLS, particularly as it pertains to cortical motor function and related pathways. Thus, TMS has garnered significant attention as a potential therapeutic modality for RLS [35]. Recent work has shown that high-frequency repetitive TMS (rTMS) can mitigate RLS symptom frequency and severity, improve reported levels anxiety and depression, and bolster sleep quality [36]. As a widely accepted method of non-invasive neuromodulation for primary sleep disorders, rTMS has been shown to be of benefit in the treatment of RLS through stimulation of bilateral primary motor cortex leg areas, left primary motor cortex at high frequency, and left primary somatosensory cortex at low frequency [22,37,38]. A significant limitation of TMS as a translatable, long-term therapy is the transient nature of its effects with symptom relapse occurring between visits. However, TMS may prove to be a reliable means of probing RLS pathophysiology with its characteristic patterns of cortical excitability, aberrant inhibitory mechanisms, and sensorimotor integration [37]. TMS has a significant amount of evidence for the safety and efficacy in studying and treating RLS; however, the transient benefit and requirement of repeated office visits for treatment administration make it less than ideal when compared to other options.

### Transcranial direct current stimulation

Transcranial direct current stimulation (tDCS) and transcutaneous spinal direct current stimulation (tsDCS) utilize low-level electrical currents, generally no greater than 1–2 mA, to mediate changes in neuronal excitability through alteration of membrane potentials [39]. With a growing body of evidence implicating SC involvement in the pathophysiology of RLS, the possibility of modifying RLS-related SC neuron excitability through tsDCS has become increasingly appealing [39, 40]. Preliminary studies have shown that tsDCS correlates with less severe RLS symptoms and improved sleep scores, but further confirmation through clinical trials is necessary to prove its efficacy [41].

### Transcutaneous electrical nerve stimulation

One trial compared transcutaneous electrical nerve stimulation (TENS) with 0.25 mg of pramipexole to pramipexole alone in a single-blinded manner to treat RLS symptoms. The TENS + pramipexole group demonstrated a superior improvement in IRLS scores and the Pittsburg Sleep Quality Index (PSQI) compared to pramipexole alone [42]. This neuromodulation technique may provide symptomatic benefit if more invasive techniques are not viable options or widely available.

### Deep brain stimulation

Studies on whether DBS for movement disorders improves RLS symptoms yield mixed findings. DBS of the subthalamic nucleus (STN) for Parkinson’s disease (PD) was first linked with RLS in 2004. However, in this case DBS unmasked RLS symptoms following the reduction of dopaminergic agonist therapy for motor symptoms of PD [43]. Further studies corroborated this initial finding, noting that patients with higher pre-DBS doses of dopamine agonists developed more debilitating symptoms of RLS post-DBS [44] The mechanism of instability of RLS symptoms in PD after DBS is unknown. A common theory is that the reduction of dopamine medication after relief of symptoms plays a role in RLS emergence while others have suggested that the microanatomy of electrode placement within the STN may determine the outcome. On the other hand, Driver-Dunckley et al. (2006) demonstrated up to 84% decrease in RLS symptoms post-STN-DBS [45]. Sex differences have also been implicated in STN-DBS for RLS, with women receiving less benefit from stimulation [46]. Other studies in STN-DBS for PD demonstrated similarly significant decreases in IRLS scores, improvements in QoL, and decreases in daytime sleepiness [47].

While all previous data consisted of case series, a systemic review of STN-DBS for RLS efficacy was demonstrated in 22 patients followed by a confirmatory meta-analysis and quantitative analyses [48–50]. Later, quantitative analysis showed that while STN-DBS may improve IRLS scores and daytime sleepiness, polysomnography parameters were not significantly affected [50]. The lack of polysomnography data improvement was confirmed with a subsequent trial; however, this trial again demonstrated improvements in IRLS scores [51]. A potential explanation for this may stem from the fundamentally unnatural environment of the test itself, illustrating the need for further studies that utilize measurements of leg movements and sleep quality in a more natural environment. The anatomical placement of contacts within the STN has only recently been studied systematically; contacts in the central sensorimotor region with contacts in the inferior sensorimotor region and substantia nigra (SN) potentially induce RLS symptoms [52,53].

Globus pallidus internus (GPi) stimulation may also provide symptomatic RLS relief. One case report of GPi-DBS in a patient with dystonia resulted in complete relief of symptoms, with symptoms recurring after one of the DBS leads was removed due to infection [54]. GPi was also the target area in the first reports of DBS for primary idiopathic RLS [55–57]. However, a pilot study using GPi-DBS failed to produce statistically significant improvements in sleep metrics [56].

Patients with essential tremor (ET) and comorbid RLS treated with DBS of the ventral intermediate nucleus (VIM) of the thalamus experienced no change in their RLS symptoms in earlier trials [58]. However, a recent study elicited the first significant response from VIM-DBS in ET patients with 5 of 13 patients experiencing complete resolution of symptoms [59].

Further prospective studies and meta-analyses analyzing the placement of the contacts during DBS can help generate treatment consensus for avoiding RLS symptom induction during the treatment of Parkinson’s using DBS. With this conflicting data, it remains unclear if this is true phenomena. Systematic, prospective outcomes data collection and analysis, particularly with evaluation of electrode placement and stimulation parameters, would be greatly informative to future investigations studying DBS to treat RLS symptoms.

### Spinal cord stimulation

SCS is a well-established treatment for multiple chronic, neuropathic pain syndromes. In our review, we have noted growing anecdotal evidence that SCS may provide effective symptomatic relief for patients with treatment-refractory RLS [60,61]. There was little data supporting SC involvement in the pathophysiology of RLS until a case report from 2016 in which the authors describe a patient receiving SCS for neuropathic pain reporting complete and durable resolution of his RLS symptoms. Specifically, this patient’s IRLS scores decreased from 33 to 0 with sustained efficacy at a 2.5-year follow-up [33]. Two other case series reported three more patients, each with comorbid RLS, experiencing relief of RLS symptoms after SCS for neuropathic pain [62,63]. A separate case reported a patient with a 44% decrease in their IRLS scores. The authors of this article suggested a mechanism behind SCS therapy improving RLS symptoms in which hypothalamic cells inhibit dopaminergic input from the spine [64]. Another case report involving two patients reported effective short-term relief of symptoms, but both patients opted to have the device removed due to lack of long-term efficacy [65]. With only case reports and case series, no significant conclusions can be drawn from the results of the studies. RCTs using SCS should be conducted in patients with primary RLS.

One case of dorsal root ganglion (DRG) stimulation (DRGS) improving RLS symptoms has been reported. The authors found a 90% reduction in reported RLS symptoms and a $90,000 cost analysis benefit when comparing SCS treatment of RLS to pharmacologic treatment [66]. The cost analysis was calculated by determining the cost of medications, procedures, and office visits the patient would pay for during the 10-year battery life of the stimulator. The cost of treatment of RLS was around $8,780 annually for treatment, which was avoided with the stimulator. As a singular case report, this study provides financial motivation toward future trials and analyses to evaluate different neuromodulatory techniques toward relief of RLS symptoms.

Further retrospective studies and subsequent meta- and qualitative analyses can inform the ultimate design of the prospective trials necessary to provide data toward these potentially life-changing therapies for patients with treatment-refractory RLS.

## Discussion

This review summarizes the current literature of neuromodulation of RLS. We compiled case reports, case series, retrospective cohort studies, and prospective trials that utilized neuromodulation modalities, including DBS, SCS, DRGS, TMS, TENS, tDCS, NPNS, and tVNS (Fig. 1). While less invasive methods, such as TMS, tsDCS, NPNS, and others did demonstrate successful symptomatic improvement, these results were transient with a decreased magnitude of effect compared to more invasive methods such as DBS and SCS. Overall, we found that DBS exhibits conflicting results with some studies showing improved symptoms while others demonstrated what seemed to be induced RLS symptoms. It has been suggested that this may be due to variation in the microanatomy within the STN and electrode placement or, alternatively, reduction in Levodopa equivalents after surgery leading to RLS symptom induction [52].

Contrarily, SCS demonstrated more consistent, promising results with greater magnitude of IRLS score improvement compared to the other modalities. In fact, some case reports show durable and complete resolution of RLS symptoms [33]. It is important to note that when reported, IRLS scores all decreased by more than 3 points, which represents the previously defined clinically significant improvement in RLS symptoms (Table 2). Unfortunately, the paucity of studies and heterogeneous reporting prevent meta-analysis of the treatment modalities necessary to provide substantial evidence supporting the use of SCS to treat RLS symptoms suggesting the need for multicenter, prospective trials (Table 3).

**Table 2:** Summary of Studies. PAG- Periaqueductal Gray, PAS- Paired Associative Stimulation, QoL- Quality of life, SN- Substantia Nigra

Author	Year	Type	Disease	Region	*N*	Type of Study	Summary
Evidente et al.	2022	DBS	ET	VIM	13	Case series	5 of 13 patients reported complete resolution of RLS symptoms after stimulation.
Ondo et al.	2006	DBS	ET	VIM	9	Case series	No effect.
Okun et al.	2005	DBS	RLS	GPi	1	Case report	Dystonia improved significantly, uncomfortable sensation and need to move resolved in left leg.
Holland et al.	2016	SCS	Pain	SC	1	Case report	Patient reported 33 to 0 decrease in RLS score after SCS therapy for neuropathic pain.
Adil et al.	2019	SCS	Pain	SC	3	Case series	3 patients reported subjective improvement of RLS symptoms after stimulation.
Byrne et al.	2019	SCS	Pain	SC	3	Case report	Three patients showed a substantial decrease in RLS scores and reduced need for pain medication.
De Vloo et al.	2019	SCS	Pain	SC	1	Case report	Case report: decrease in IRLS score by 44% and RLS 6-item questionnaire scores by 25%, resulting in 33% improvement in RLS-QoL questionnaire scores
Klepitskaya et al.	2018	DBS	PD	STN	22	Case series	Statistically significant Class IV evidence supporting use of STN for RLS in PD patients.
Chahine et al.	2011	DBS	PD	STN	6	Case series	Significant decrease in RLS score and 4 weeks and 6 months (*p* = 0.027, *p* = 0.037), less daytime sleepiness, higher QOL.
Driver-Dunckley et al.	2006	DBS	PD	STN	6	Case series	RLS scores dropped by a mean of 84% in all participants after stimulation and 100% in three participants.
Dulski et al.	2022	DBS	PD	STN	36	Case series	Improvement in RLS scores and survey of symptoms, resolution of RLS in 43% of participants, but no change in polysomnography parameters.
Hidding et al.	2019	DBS	PD	STN, SN	15	RCT	STN + SN stimulation was comparable to STN stimulation in most situations and was superior to STN regarding RLS symptoms experienced at night.
Kedia et al.	2004	DBS	PD	STN	11	Case series	Reduction of parkinsonian medication may unmask RLS during DBS-STN
Lei et al.	2022	DBS	PD	STN	363	Retrospective clinical study	Activated contacts that relieved RLS were in the central sensorimotor region of the STN while contacts in the inferior sensorimotor and SN may have induced RLS; DBS-STN improved RLS in patients with PD in one year
Marques et al.	2015	DBS	PD	STN	31	Case series	Emergence of RLS after STN stimulation occurred in 6/31 patients, with those patients usually having higher dopamine agonist usage.
Tordjman et al.	2024	DBS	PD	STN	1	Case report	Symptoms of RLS were induced by STN DBS for PD and were not temporally associated with dopaminergic medication and disappeared when DBS was deactivated.
Tolleson et al.	2016	DBS	PD	GPi	5	Prospective clinical trial	Surveys showed improvement with ISI scale showing a promising trend post pallidal stimulation, sleep latency, and efficiency after pallidal stimulation improved but not to a statistically significant degree.
Abd-Elsayed et al.	2024	NPNS	RLS	DRG	1	Case report	90% reduction in reported symptoms with a 10-year cost analysis savings of $90,000.
Altunrende et al.	2014	rTMS	RLS	Supplementary motor area	19	RCT	rTMS significantly improved IRLS-RS scores while the sham stimulation did not.
Ahlgren-Rimpilainen et al.	2012	TMS	RLS	Primary motor cortex	12	Case series	RLS group exhibited more CSPs in dominant ADM, TA, and nondominant ADM versus controls.
Bocquillon et al.	2017	TMS, NPNS	RLS	Primary motor cortex, median nerve	28	Case series	ANOVA showed main effect of ISI and group and showed a group x ISI interaction while no difference was found for SAI and LAI ISIs.
Bogan et al.	2023	NPNS	RLS	Common peroneal nerve	133	RCT	Significant improvement in CGI-I (28%) and RLS scores were achieved in patients with TOMAC NPNS with no serious adverse effects.
Buchfuhrer et al.	2021	NPNS	RLS	Common peroneal nerve	37	RCT	NPNS group reported a significant 6.8-point improvement on the IRLS scale compared to 3.4-point improvement by sham. Also, significant increase in CGI-I. There were no differences in subgroup analyses between medication resistant and medication naïve patients.
Buchfuhrer et al.	2023	TOMAC	RLS	Common peroneal nerve	20	Case series	TOMAC significantly reduced the amount of opioid use for RLS by 20% in 70% of study participants while maintaining CGI-I score under or at 5, suggesting the potential for TOMAC to reduce opioid use by RLS patients.
Casoni et al.	2020	DBS	RLS	GPi	1	Case report	Patient showed both subjective improvement through the RLS survey and objective improvement through polysomnography parameters.
de Paiva et al.	2017	rTMS	RLS	Primary motor cortex	57	Case series	Significant decreases in CSP duration occurred but there were no differences in CSP duration between subjects with RLS versus controls.
Gorsler et al.	2007	TMS	RLS	Primary motor cortex	17	Case series	Patients showed significant CSP shortening compared to healthy subjects before cabergoline treatment. After 14 days of treatment, CSP normalized in RLS patients but shortened again after 90 days of daily cabergoline. RLS symptoms improved.
Gunduz et al.	2012	rTMS	RLS	Primary motor cortex	19	Case series	Cortical active motor threshold, but not other parameters, was significantly lower in the RLS group than the control group at night (28.5 ± 6.2% vs. 40.4 ± 8.4%).
Hackethal et al.	2023	SCS	RLS	SC	2	Case series	Both patients reported short term benefits, with only one of the patients reporting long term (6 mo) benefits. However, both had their devices removed due to inefficacy.
Hartley et al.	2023	tVNS	RLS	Left cymba concha	15	Case series	Significant Improvement in RLS scores, QoL, anxiety and depression.
Klung et al.	2020	TMS	RLS	Leg motor area	1	Case report	Patient reported noticeable improvement in RLS 1 month after treatment citing decreased medication reliance.
Kutukcu et al.	2006	TMS	RLS	Primary motor cortex	35	Case series	CSP duration was significantly shorter in-patient group for both ABP and TA. After 1-month drug administration, thresholds and CSP, measured bilaterally from the APB, showed no significant difference, but CSP durations for the TA muscles showed a significant prolongation.
Lanza et al.	2018	rTMS	RLS	Primary motor and somatosensory cortices	23	Case series	CSP was shorter in RLS versus control groups and remained shorter for both motor and somatosensory stimulation. Subjective improvement was found in the RLS group in initiating and maintaining sleep. Sham was ineffective.
Lin et al.	2015	rTMS	RLS	Primary motor cortex	14	Case series	Significant improvement in IRLS-RLS (23.86 ± 5.88 to 11.21 ± 7.23), PSQI (15.00 ± 4.88 to 9.29 ± 3.91), and HAMA (17.93 ± 7.11 to 10.36 ± 7.13) scale scores with effects lasting 2 months.
Lin et al.	2018	TMS	RLS	Primary motor cortex	26	Case series	No significant differences in RMT or H-reflex latencies or amplitudes were found between RLS vs. control. Significant increase in unconditioned MEP amplitudes of TA was observed in patients compared to controls. LAI of median nerve in RLS patients was significantly decreased at IAIs of 150 and 200 ms.
Liu et al.	2015	TMS	RLS	Primary motor cortex	29	Case series	RLS patients showed lower ALFF in the sensorimotor and visual processing regions than healthy controls and high ALFF in the insula, parahippocampal and hippocampal gyri, left posterior parietal areas, and brainstem. ALFF in several sensorimotor and visual regions were significantly elevated and IRLS Rating Scale scores decreased after rTMS.
Magalhaes et al.	2019	TMS	RLS	Primary motor cortex	54	Case series	SICI was significantly reduced in patients with mild to moderate and severe to very severe RLS versus controls.
Ondo et al.	2012	DBS	RLS	GPi	1	Case report	Patient had positive response, but far from complete that affected the urge to move and involuntary movements more than pain.
Quatrale et al.	2003	TMS	RLS	Primary motor cortex	27	Case series	Short ISI paired TMS significantly decreased inhibition and increase in facilitation in ADM muscles and result was more evident in TA muscles compared to controls, modifications were more evident in limbs more affected by PLM.
Rizzo et al.	2009	TMS	RLS	Primary motor cortex	22	Case series	PAS significantly increased corticospinal excitability by 30 min in healthy subjects. PAS did not change MEP amplitudes in patients with idiopathic RLS without treatment.
Rizzo et al.	2010	TMS	RLS	Primary motor cortex	20	Case series	SAI was significantly reduced in RLS patients compared to control. Dopaminergic treatment normalizes alteration of sensory-motor integration.
Roy et al.	2023	TOMAC	RLS	SCS	103	RCT	CGI-I responder rate increased from 63.3% at RESTFUL completion to 72.7% at week 24 for treatment group versus 13.6% at week 24 for control group. Mean change in IRLS score improved from − 7.4 at RESTFUL completion to -11.3 points at week 24 for treatment group versus − 5.4 at week 24 for control group. TOMAC significantly maintained safety and efficacy with usage up to 24 weeks, with some symptomatic maintenance after cessation of stimulation.
Salas et al.	2018	TMS	RLS	Primary motor cortex	66	Case series	No significant differences in baseline rMT between RLS and control groups in M1 hand, rMT for leg TA was significantly lesser for RLS than controls.
Sanli et al.	2024	TENS	RLS	Dorsiflexor group and extensor hallucis longus muscle	46	RCT	Combination therapy of TENS unit and pramipexole provided therapeutic benefit reported through increased IRLS and PSQI scores compared to pramipexole alone.
Scalise et al.	2006	TMS	RLS	Primary motor cortex	11	Case series	CSP duration was shorter in RLS patient’s vs. controls.
Scalise et al.	2010	TMS	RLS	Primary motor cortex	24	Case series	MEP amplitude increased insignificantly in RLS patients after resting post-treatment showing delayed facilitation, and after exercise showing positive but not clear post-exercise facilitation. Central motor inhibition was increased in RLS group. Duration of SP did not change compared to pre-treatment condition.
Singh et al.	2024	TOMAC	RLS	Common peroneal nerve	45	RCT, meta-analysis	IRLS reduction was significantly greater for TOMAC than sham (-6.59 vs. -2.17) confirming safety and long-term efficacy of previous trial. Performed meta-analysis and found that TOMAC significantly improves RLS symptoms in naïve participants.
Tergau et al.	1999	TMS	RLS	Primary motor cortex	35	Case series	Intracortical inhibition paired with TMS was significantly reduced for both foot and hand muscles.
Zeng et al.	2020	tsDCS	RLS	SC	50	Case series	RLS groups showed significantly decreased IRLS-RS (*p* < 10^-6) and PSQI (*p* < 10^-4) scores after tsDCS compared with before treatment. Sham tsDCS showed no significant changes in IRLS-RS and PSQI scores.

**Table 3:** Effect of SCS on IRLS Scores. IRLS-International Restless Legs Syndrome Scale. *Calculated from percentage reported in article. #Did not report IRLS score, only on a scale 1–10

Citation	*N*	D IRLS Scores
Holland et al. 2016	1	-33
Adil et al. 2019^#^	3	10/10→ 1/10
		-
		9/10→ 1/10
Byrne et al. 2019	3	-9
		-12
		-29
De Vloo et al. 2019^*^	1	-16

**Fig. 3:**
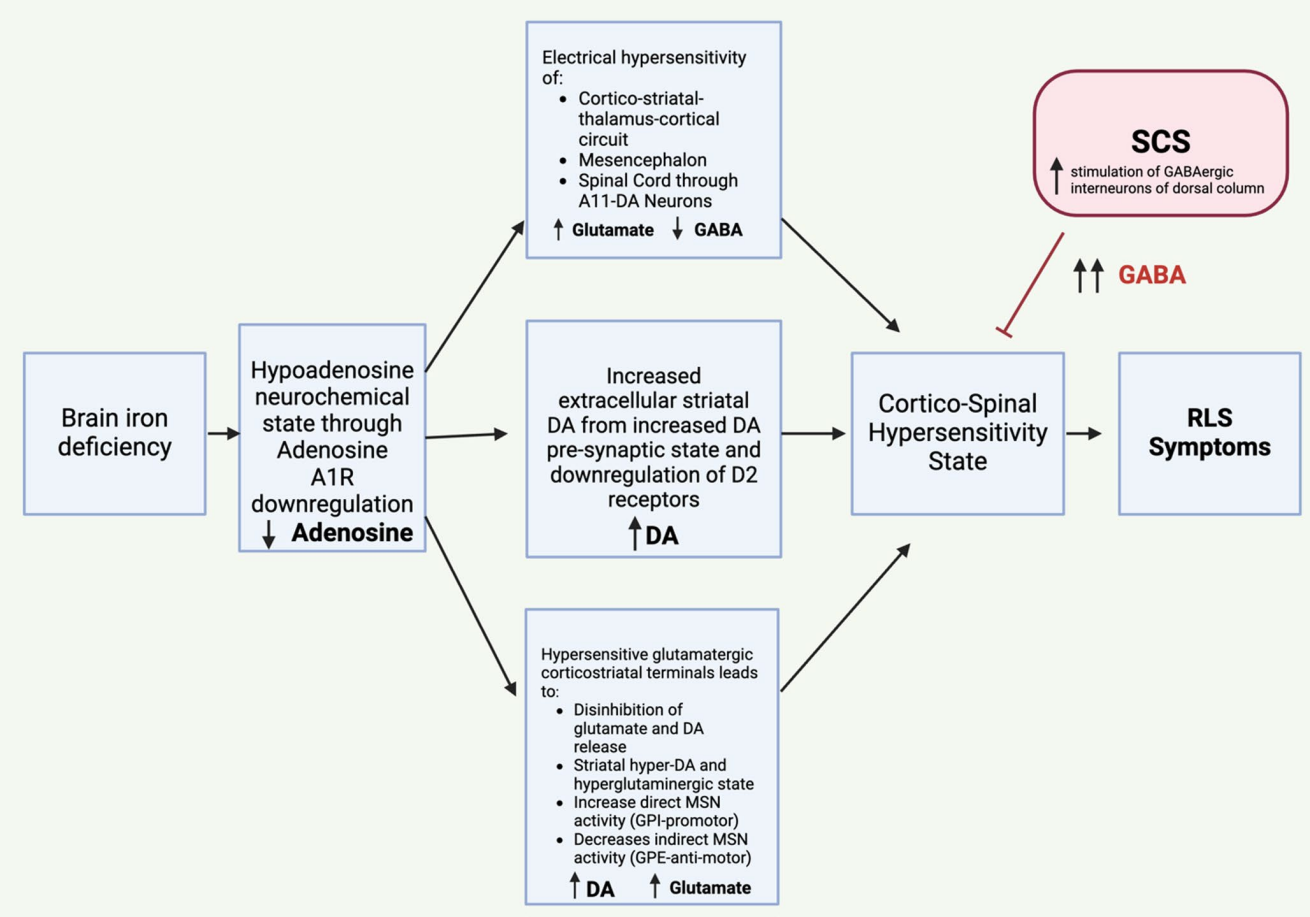
Proposed mechanism of action of spinal cord stimulation (SCS) in restless legs syndrome (RLS) by a hypersensitive cortico-spinal state. DA – Dopamine

The pathophysiological mechanism behind SCS for RLS is unclear, but evidence suggests abnormalities in the dopaminergic, serotonergic, and adrenergic pathways with a resultant central nervous system (basal ganglia, cortex, and SC) hypersensitivity [67]. As patients experience circadian cycling of their symptoms, so too do these neurochemicals and neuroelectrophsyiological circuitries [68]. Specifically, these symptoms seem to be inversely coordinated with dopamine release [69]. Lack of dopamine in the basal ganglia, cortex, and SC results in a hypersensitive central nervous system state [70]. This hypersensitivity creates a dysfunctional sensory-motor integration condition in the SC and cortex (Fig. 3)[71]. This dysfunctional integration may help explain RLS symptoms with the inability to filter the “urge” percept at the SC and cortex. The lack of appropriate sensory-motor integration could additionally help explain the involuntary leg movements displayed by RLS patients. In fact, some studies provide evidence of improper SC network modulation with an abnormal H-reflex, the electrical equivalent of a deep tendon reflex, recorded from RLS patients [16]. If the SC is hypersensitive and unable to adequately filter the incoming signal, an involuntary leg movement may result.

The origin of the “urge” percept in RLS is one of much interest and speculation. Patients often report the “urge” originating from within the limbs, with relief from the visceral feeling only relieved by muscle contraction or getting up and walking. We hypothesize that the urge sensation may be generated by hyperactive Type Ia, Type Ib, and/or Type II fibers that originate in the muscle spindle and Golgi tendon organ [72]. These fibers synapse in the dorsal horn of the SC that directly assist in the stretch reflex (deep tendon reflex), relaxing of the muscle tension, as well as providing proprioceptive information to the central nervous system are important for maintaining posture and coordination [73]. The ascending information is carried through large, myelinated Aα fibers in the dorsal column. Should the dorsal horn of the SC be in a hypersensitive state (evening, low dopamine), it is reasonable to speculate that this information may not be properly processed and result in abnormal, involuntary movements. Additionally, the ascending fibers may carry this abnormal percept to the cortex where it could be interpreted by a hyperexcitable cortex with impaired sensory-motor integration as “urge.”

DBS may directly modulate or improve basal ganglia functional output to decrease cortical hyperexcitability [74]. TMS may increase local GABA in the hyperactive cortex [75]. However, TMS has the disadvantage of requiring repeated, in-clinic treatments due to its transient effects. TMS represents a valuable tool for further investigations into the pathophysiology and biological plausibility of effectiveness given its ability to directly evaluate cortical hypersensitivity. DRGS, TENS, tDCS, and NPNS may increase Aβ fiber activity, although evidence suggests that their effects on RLS symptoms are not as robust.

We believe that SCS currently represents the most intriguing, viable neuromodulation therapy for RLS that demands further investigation. We hypothesize that SCS could interrupt this “urge” signal in multiple ways. First, it is possible that the SCS is directly modulating the signal traveling in the Aα neurons of the dorsal column that could block the “urge” percept prior to it arriving at higher brain centers [76]. Additionally, the gate control theory of pain that underlies the current biological principal theory of SCS effectiveness dictates that the dorsal column Aβ fibers that carry light touch information, through their normal orthodromic conduction, synapse upon an inhibitory interneuron in the spinal cord that further synapses within the dorsal horn [77]. Stimulation of Aβ fibers, and, thus, inhibitory spinal interneurons, leads to increased GABA within the dorsal horn and increased inhibition of incoming sensory signals, potentially including hyperactive proprioceptive signals [78]. With its biological plausibility, preliminary effectiveness demonstrated in prior reports, and long safety record, we believe that SCS represents a strong risk to benefit ratio for further prospective investigations. It is also important to note that SCS is less invasive than DBS, not requiring penetration of the dura. It is fair to speculate that patients may be more receptive to this neuromodulation modality even without treatment-refractory symptoms, similar to PD patients undergoing DBS with the goal of medication reduction.

## Limitations

Multiple limitations exist in this review that raise questions regarding the study’s quality. Publication bias exists as it is intrinsic to systematic reviews. This protocol was also not registered in a database before beginning the review, which poses a risk of duplication of study type and topic. Heterogeneity among the studies included in this review is a critical limitation as different protocols and different patient populations produce results that may not be comparable among each other. The statistically significant effects of each neuromodulation modality were unable to be evaluated due to the heterogeneity in follow-up, outcomes reported, and lack of data granularity limiting the article to observational anecdotes and summaries of the results. Despite this, the degree of RLS score recovery seems to positively correlate with the degree of invasiveness of a given procedure among the studies included in this review. It is important to acknowledge that expectation bias may exist within increasingly invasive neuromodulatory treatment; increased invasiveness of a treatment may lead to higher expectations for resolution of symptoms due to increased need for hardware, software, increased procedure time, increased procedure complexity, and more, which can produce a placebo effect. The methodological quality of the studies included in this review also contain risk of bias; While some of the studies were RCTs, many were also case series or case reports which have a higher risk of bias due to their uncontrolled nature whereas RCTs control for as many potential confounding variables as possible [79]. Lastly, many studies investigating neuromodulation to treat RLS symptoms include patients that have RLS concomitant to another pathology with very few patients having RLS as their primary indication for neuromodulation. Many studies included in this review examine the effects of neuromodulation on secondary RLS as opposed to primary RLS. This is important to acknowledge as comorbid RLS differs to primary RLS. This may result in limited applicability or translatability of this review’s conclusions and may result in inaccurate generalizations under the assumption that therapies are directly targeting RLS symptoms rather than resolution of RLS symptoms being a secondary outcome of treating the concomitant disorder that is not RLS. Studied observing effects of neuromodulation in patients with primary RLS should be conducted.

## Conclusion

Without rigorous statistical analyses from prospective trials of the multiple neuromodulatory techniques, discerning the most effective form of neuromodulation for an individual patient remains challenging. In treatment-refractory PD, treatment selection is based upon several factors, such as concomitant sequelae, disease burden, and invasiveness of the procedure. In many cases, patients with debilitating PD who have comorbid RLS undergo DBS treatment primarily for their Parkinson’s symptoms. More intriguing, however, is the patient with primary idiopathic RLS for which little data in the more invasive treatments (i.e., SCS, DBS) exist. Comparison across the newer, less invasive trials may be useful for less severe cases of treatment-refractory RLS, or for use supplemental to medical treatment. Based on the literature available, the degree of RLS score recovery correlates with the procedure’s invasiveness, with the highly invasive modalities including DBS and SCS providing the most symptom relief. Furthermore, SCS has been shown to completely reduce subjective intensity of RLS symptoms for up to 2.5 years and, overall, demonstrate more consistent, promising results with greater magnitude of RLS symptoms resolution in comparison to other neuromodulatory methods. We propose utilizing the IRLS, CGI-I, and objective measures of sleep and leg movements, preferably in the ambulatory setting, as key measures in these trials. The sequential trials and analyses must control for treatment modalities, severity of disease, invasiveness of procedure, and anatomical location/optimization of the invasive procedures to develop guidelines and personalized treatment plans. Further prospective, controlled, multi-center, multi-modality trials studying the feasibility, safety, and efficacy of each neuromodulatory modality are necessary due to the heterogeneity of the data and methods of each study, and to provide significant evidence to guide treatment of treatment-refractory RLS using neuromodulation.

## Data Availability

The authors confirm that the data supporting the findings of this study are available within the article and its supplementary materials.

## Abbreviations

CGI-I: clinical global impressions-improvement
DBS: deep brain stimulation
DRG: dorsal root ganglion
DRGS: dorsal root ganglion stimulation
ET: essential tremor
GPi: globus pallidus internus
IRLS: International Restless Legs Scale
NPNS: noninvasive peripheral nerve stimulation
PD: Parkinson's disease
PSQI: Pittsburg Sleep Quality Index
QoL: quality of life
RLS: restless legs syndrome
rTMS: repetitive transcranial magnetic stimulation
SC: spinal cord
SCS: spinal cord stimulation
SN: substantia nigra
STN: subthalamic nucleus
tDCS: transcranial direct current stimulation
TENS: transcutaneous electrical nerve stimulation
TMS: transcranial magnetic stimulation
TOMAC: tonic motor activation
tsDCS: transcutaneous direct current stimulation
tVNS: transauricular vagus nerve stimulation
VIM: ventral intermediate nucleus
